# Blockade of NLRP3/Caspase-1/IL-1*β* Regulated Th17/Treg Immune Imbalance and Attenuated the Neutrophilic Airway Inflammation in an Ovalbumin-Induced Murine Model of Asthma

**DOI:** 10.1155/2022/9444227

**Published:** 2022-05-25

**Authors:** Ling Chen, Weiwei Hou, Fen Liu, Ruochen Zhu, Anping Lv, Wenqiang Quan, Song Mao

**Affiliations:** ^1^Department of Pediatrics, Shanghai Jiao Tong University Affiliated Sixth People's Hospital, Shanghai 200233, China; ^2^Department of Laboratory Medicine, Shanghai Tongji Hospital, School of Medicine, Tongji University, Shanghai 200065, China

## Abstract

Asthma is a heterogeneous inflammatory disorder of the airways, and multiple studies have addressed the vital role of the nucleotide-binding oligomerization domain-like receptor family pyrin domain containing 3 (NLRP3)/caspase-1/interleukin-1*β* (IL-1*β*) pathway in asthma, but its impact on ovalbumin- (OVA-) induced neutrophilic asthma remains unclear. Here, we explored this pathway's effect on airway inflammation in neutrophilic asthma to clarify whether blocking this signaling could alleviate asthmatic airway inflammation. Using an established OVA-induced neutrophilic asthma mouse model, we provided asthmatic mice with a highly selective NLRP3 inhibitor, MCC950, and a specific caspase-1 inhibitor, Ac-YVAD-cmk. Our results indicated that asthmatic mice exhibited increased airway hyperresponsiveness, neutrophil infiltration, and airway mucus hypersecretion, upregulated retinoid-related orphan receptor-*γ*t (ROR*γ*t) mRNA expression, and downregulated fork head box p3 (Foxp3) mRNA expression, which was concurrent with NLRP3 inflammasome activation and upregulation of caspase-1, IL-1*β*, and IL-18 expression in lung. Treatment of NLRP3 inflammasome inhibitors significantly attenuated airway hyperresponsiveness, airway inflammation, and reversed T helper 17 (Th17)/regulatory T (Treg) cell imbalance in asthmatic mice. We propose that the NLRP3/caspase-1/IL-1*β* pathway plays an important role in the pathological process of neutrophilic asthma and provides evidence that blocking this pathway could potentially be a treatment strategy to ameliorate airway inflammation in asthma after validation with future experimental and clinical studies.

## 1. Introduction

Bronchial asthma, referred to as asthma, is a heterogeneous disease with cough, chest tightness, wheezing, shortness of breath, and other respiratory symptoms as the main clinical manifestations. Asthma is an inflammatory disease with airway hyperresponsiveness (AHR) and reversible airway obstruction [1, 2]. There are about 310 million bronchial asthma patients worldwide. With the continuous deterioration of air quality and environmental conditions, the incidence of bronchial asthma is increasing year by year [3, 4]. In current clinical treatment, corticosteroids and inhaled bronchodilators are mainly used to improve asthma; however, they may also produce undesired side effects, as well as patient resistance and unresponsiveness to long-term use. Therefore, there is an urgent need to find new and more specific asthma therapeutic targets in clinical treatment, especially for neutrophil subtypes of severe asthma. This subtype is mainly characterized by neutrophil-dominant airway inflammation, increased Th17-mediated immune responses, and less response to corticosteroids [5, 6].

Recently, a growing number of studies have looked at the role of inflammasomes in airway diseases. The NLRP3 inflammasome is an intracellular sensor that detects damage-associated molecular patterns (DAMPs) and pathogen-associated molecular patterns (PAMPs) and represents a crucial component of innate immune responses in the airways [7, 8]. Activated NLRP3 binds to the apoptosis-associated speck-like protein containing CARD (ASC), which in turn interacts with the cysteine protease caspase-1 to form a complex called the inflammasome, further leading to caspase-1 activation. Caspase-1 further cleaves the proinflammatory cytokines IL-1*β* and IL-18 into their active forms [9, 10]. IL-1*β* is a well-recognized inducer of neutrophilia potentially contributing to the pronounced airway inflammation [11]. IL-18 responds to external factors, triggers lots of proinflammatory reactions, and participates in hyperreactivity of the respiratory tract [12]. In addition, recent evidence indicated that IL-1*β* and IL-18 act in synergy with IL-23 to promote the differentiation of Th17 cells and IL-17A production that contributes to AHR and neutrophilic airway inflammation [13, 14]. Accumulating evidence has demonstrated that NLRP3 inflammasome is involved in the pathogenesis of airway inflammation and the process of airway remodeling in asthma [15, 16]. Mice with allergic asthma induced by OVA and aluminium adjuvants demonstrated enhanced protein expression of NLRP3 and caspase-1, along with elevated IL-1*β* and IL-18 release by epithelial cells and macrophages in the airways [17]. Furthermore, in murine models of asthma induced by OVA or house dust mite (HDM), blocking NLRP3 with specific inhibitor or gene knockout mice significantly alleviated asthma symptoms [18, 19].

Given this background, the NLRP3/caspase-1/IL-1*β* pathway apparently plays an important role in the pathogenesis of asthma, but the precise effects of NLRP3 inflammasome activation on airway inflammation in an OVA-induced murine model of neutrophilic asthma remain elusive and deserve investigation. In our study, female Balb/c mice were used to establish the neutrophilic asthma model and simultaneously administered with a high selective NLRP3 inhibitor, MCC950, as well as the specific caspase-1 inhibitor Ac-YVAD-cmk for therapeutic purposes, and the relationships among NLRP3/caspase-1/IL-1*β* pathway, neutrophilic airway inflammation, and Th17/Treg immune responses were determined.

## 2. Materials and Methods

### 2.1. Animals

Six to eight-week old female Balb/c mice (16-18 g) were obtained from Shanghai Institute of Planned Parenthood Research (Shanghai, China). Twenty-four mice of the same strain and sex, with similar age and weight, were divided into four groups according to the complete randomization method, and each group contained six animals. Mice were bred and housed in a specific pathogen-free (SPF) laboratory with 12 hr light-dark cycle and 24-26°C ambient temperature. Physiological status of mice was checked 3 times a week before OVA sensitization and daily after sensitization. Overdose of sodium pentobarbital by intraperitoneal injection was used before sacrifice in order to minimize suffering and distress. No seriously ill or dead mice were found prior to sacrifice. All experiments were approved by Institutional Animal Care and Use Committee of Shanghai Jiao Tong University, Shanghai, China (Permit Number: DWSY2020-0074).

### 2.2. OVA-Induced Asthma and Interventions

**Figure 1:**
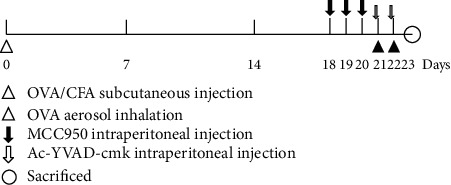
Ovalbumin sensitization, challenge, and intervention protocol. Balb/c mice were systemically sensitized by subcutaneous injection with OVA/CFA on day 0 and then challenged with OVA aerosols on days 21 and 22. MCC950 was administrated into mice by intraperitoneal injections for three consecutive days before the aerosols. Three hours before each exposure to aerosol, mice were injected intraperitoneally with Ac-YVAD-cmk. OVA: ovalbumin; CFA: complete Freund's adjuvant.

Mice were divided randomly into four groups: control, neutrophilic asthma (NA), MCC950, and Ac-YVAD-cmk (A-Y-c), and each group contained six mice. Control group: mice were injected subcutaneously with phosphate-buffered saline (PBS) on day 0 and then inhaled PBS by nebulizer for 20 minutes on days 21 and 22; NA group: mice were sensitized by subcutaneous injections with 20 *μ*g of grade V chicken egg OVA (Sigma-Aldrich), emulsified in 75 *μ*l complete Freund's adjuvant (CFA, Sigma-Aldrich) on day 0, and then exposed to aerosols consisting of 1% OVA on days 21 and 22; MCC950 group: mice were given daily intraperitoneal injections of MCC950 (50 *μ*g/g, Sigma-Aldrich) for three consecutive days before the aerosols; A-Y-c group: Ac-YVAD-cmk (5 *μ*g/g, Sigma-Aldrich) was administrated into mice by intraperitoneal injections three hours before each aerosol inhalation (Figure 1).

### 2.3. Assessment of Hyperresponsiveness

Twenty-four hours after the last challenge, pulmonary resistance measurements were performed in anesthetized and mechanically ventilated mice in response to increasing doses of methacholine(Sigma-Aldrich) by ultrasonic nebulization (3.125, 6.25, 12.5, 25, and 50 mg/ml), as described in previous research [20]. Pulmonary resistance measurements were performed every five minutes after each nebulization step until a plateau was reached. The resistance index (RI) for each methacholine concentration represents the results.

### 2.4. Bronchoalveolar Lavage Fluid (BALF) Collection and Differential Cell Counts

Mice were sacrificed by overdose of sodium pentobarbital and bronchoalveolar lavage (BAL) was performed after the assessment of hyperresponsiveness. Briefly, the trachea was cannulated, and the lungs were lavaged 3 times with PBS. The collected BALF was centrifuged to obtain a pellet containing BAL cells and supernatant. Gently dissolve the pellet in 200 *μ*l PBS and store the supernatant at -80°C for cytokine analysis. Differential cell counts of at least 300 cells were performed according to standard morphological criteria on Wright-Giemsa (Beyotime, China) stained slides. Cell numbers were counted and expressed as mean ± SEM per milliliter per group.

### 2.5. Myeloperoxidase (MPO) Activity Measurement

Measurement of lung MPO activity is to assess neutrophil inflammation as described in earlier studies [21]. Briefly, thiobarbituric acid was added to BALF or lung tissue homogenate, and the mixture was centrifuged to take the supernatant, which was measured spectrophotometrically to assess MPO activity.

### 2.6. Enzyme-Linked Immunosorbent Assay (ELISA)

Cytokine concentrations in BALF were measured using specific mouse IL-1*β*, IL-18, IL-17A, and IL-10 ELISA kits (eBioscience). The experimental method was performed strictly according to the manufacturer's instructions.

### 2.7. Western Blotting

Lung tissues were homogenized with ice-cold radioimmunoprecipitation assay buffer (Beyotime, China) containing protease inhibitors and separated on a 12% SDS-polyacrylamide gel. In the next step, the membranes are transferred to polyvinylidene fluoride (PVDF) membranes and then blocked with 5% nonfat milk in a Tween 20 buffer. The membranes are then incubated with the following primary antibodies: rat anti-mouse NLRP3 (2 *μ*g/ml, R&D) and goat anti-mouse IL-1*β* (0.25 *μ*g/ml, R&D). After incubating the samples overnight, the corresponding horseradish peroxidase-conjugated anti-rat or anti-goat IgG secondary antibodies (1/10000 dilution, R&D) were added. Band signals were visualized by enhanced chemiluminescence using an ECL development kit (Beyotime, China) according to the manufacturer's instructions.

### 2.8. RT-qPCR Analysis

After the lungs were homogenized, total RNA was extracted using Trizol reagent (Invitrogen), and the quality of extracted RNA was analyzed using an Agilent 2100 Bioanalyzer (Agilent Technologies). cDNA samples were obtained by reverse transcription reaction using PrimeScript Reverse Transcriptase (TaKaRa). ABI Prism 7500 (Applied Biosystems) was used with the following program to perform real-time PCR: 95°C for 2 min, 95°C for 15 s, 40 cycles of amplification, 58°C for 15 s, and 72°C for 60 s. All primer sequences were synthesized by Shanghai Biosune Biotechnology Company. Primer were as follows: GAPDH forward 5′-TGA ACC ACG AGA AAT ATG ACA AC-3′, reverse 5′-ATG AGC CCT TCC ACA ATG C-3′; NLRP3 forward 5′-CCT GGT CTG CTG GAT TGT G-3′, reverse 5′-AGT GGT CTT GGA GGT CTG G-3′; caspase-1 forward 5′-ATC TTT CTC CGA GGG TTG G-3′, reverse 5′-AAG TCT TGT GCT CTG GGC AG-3′; IL-1*β* forward 5′-TTC AGG CAG GCA GTA TCA C-3′, reverse 5′-CAG CAG GTT ATC ATC ATC ATC C-3′; IL-18 forward 5′-ACT GTA CAA CCG CAG TAA TAC-3′, reverse 5′-AGT GAA CAT TAC AGA TTT ATC CC-3′; ROR*γ*t forward 5′-ATT CAG TAT GTG GTG GAG TTT G-3′, reverse GTG GTT GTT GGC ATT GTA GG-3′; and Foxp3 forward 5′-CCA GGA CAG ACC ACA CTT C-3′, reverse 5′-CGC ACT TGG AGC ACA GG-3′. GAPDH gene expression was used as a reference for data normalization. The fold change was calculated by the 2^-△△CT^ method.

### 2.9. Flow Cytometry (FCM)

Mice spleens were isolated, and cell clumps were disintegrated and filtered using nylon mesh (70 *μ*m pore size) to form a single cell suspension. 1× RBC lysis buffer (eBioscience) was used to remove the erythrocytes. In order to detect Th17 and Treg cells, cells were treated with a lymphocyte activator mixture (50 ng/ml phorbol 12-myristate 13-acetate (PMA, Sigma-Aldrich), 1 *μ*g/ml ionomycin (Peprotech), and 1/1000 dilution brefeldin A (eBioscience)) for five hours and labeled with surface markers FITC anti-CD4 mAb (eBioscience) and APC anti- CD25 mAb (eBioscience). Cells were washed, fixed, and permeabilized based on instructions from the manufacturer (eBioscience) and then stained intracellularly with PE-Cyanine7 anti-IL-17 mAb (Biolegend) or PE-anti-Foxp3 mAb (eBioscience). We electronically gated lymphocytes according to their forward scatter and side scatter properties, and then, we detected all labeled cells using FCM on our CytoFLEX Flow Analyzer. The data were analyzed using the CytExpert software (version 2.4).

### 2.10. Lung Histopathological Analysis

After collecting BALF, the low right lobe of the lungs was preserved in 4% buffered formaldehyde and paraffin embedded. Lungs were cut into 4 *μ*m slices and stained with H&E and PAS to evaluate histological alterations. Three observers independently scored peribronchial inflammatory infiltration (0-5) as follows [22]: 0, normal; 1, a few cells; 2, a ring of inflammatory cells 1 cell layer deep; 3, a ring of inflammatory cells 2 to 4 cells deep; and 4, a ring of inflammatory cells > 4 cells deep. The five points of goblet cell hyperplasia in the airway epithelium are as follows: 0, normal; 1, <25% of the epithelium; 2, 25-50% of the epithelium; 3, 50-75% of the epithelium; and 4 equaled >75% of the epithelium [23].

### 2.11. Statistical Analysis

GraphPad Prism 8.3.1 was used for graphing and statistical analysis. Data were given as mean ± SEM, and group comparisons were made using one-way ANOVA with Bonferroni post hoc tests for multiple comparisons. A value of *p* < 0.05 was considered statistically significant.

## 3. Results

### 3.1. Blockade of the NLRP3/Caspase-1/IL-1*β* Pathway Ameliorated Airway Hyperresponsiveness, Neutrophilic Airway Inflammation in OVA-Induced Asthma

**Figure 2:**
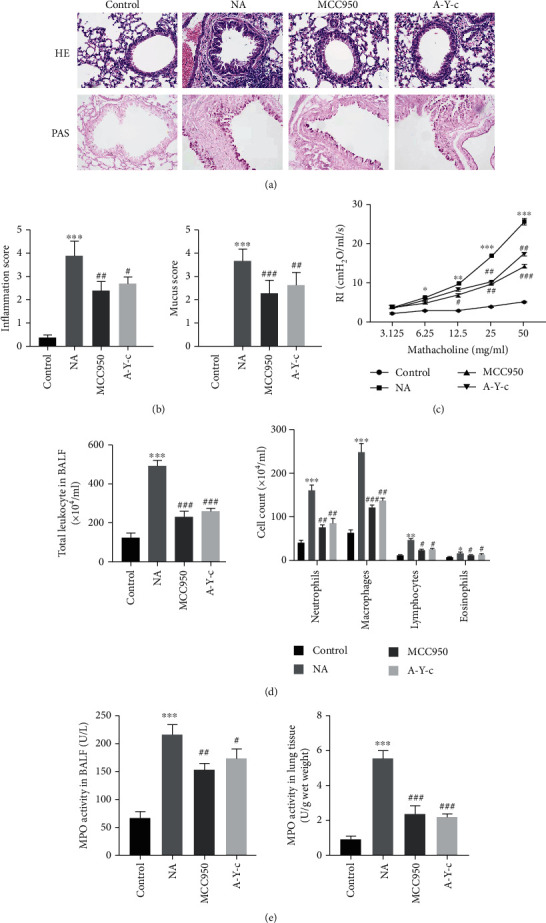
Blockade of the NLRP3/caspase-1/IL-1*β* pathway ameliorated airway hyperresponsiveness, airway inflammation induced by ovalbumin. (a) Representative H&E-stained and PAS-stained lung sections of different groups. Original magnification was 200x. (b) Semiquantification of airway inflammation and PAS staining was performed. (c) Resistance index (RI) to double multiplication concentration of methacholine (3.125, 6.25, 12.5, 25, and 50 mg/ml) was measured. (d) Number of total inflammatory cells, neutrophils, macrophages, lymphocytes, and eosinophils in BALF. (e) MPO activity in BALF and lung tissue. There were six mice in each group. Data are expressed as mean ± SEM. Compared to the control group, ^∗^*p* < 0.05, ^∗∗^*p* < 0.01, and ^∗∗∗^*p* < 0.001; compared to the neutrophilic asthma group, ^#^*p* < 0.05, ^##^*p* < 0.01, and ^###^*p* < 0.001. NLRP3: nucleotide-binding oligomerization domain-like receptor family pyrin domain containing 3; MPO: myeloperoxidase; BALF: bronchoalveolar lavage fluid.

To explore the effects of the NLRP3/caspase-1/IL-1*β* pathway on asthmatic airway inflammation, we established the murine model of neutrophilic asthma induced by OVA combined with CFA. As indicated in Figures 2(a) and 2(b), the NA group had significantly more bronchial inflammation, epithelial goblet cell hyperplasia, and mucus hypersecretion than the control group, which alleviated after MCC950 therapy. Aligned with this finding, OVA-challenged mice exhibited significantly increased airway reactivity to methacholine accompanied by increased inflammatory cell infiltration compared to the control group, and the difference was statistically significant (*p* < 0.05), including neutrophils, macrophages, lymphocytes, and eosinophils into the airways. This reaction was reversed with MCC950 treatment (Figures 2(c) and 2(d)).

To further explore the function of neutrophils, the MPO activity was measured, and the data showed that its activity in both BALF and lungs from the asthma group was significantly increased versus the control group (both *p* < 0.05), and these increases were both markedly reversed by MCC950 (Figure 2(e)). Meanwhile, caspase-1 inhibitor Ac-YVAD-cmk was also administrated into mice before aerosol inhalation, and similar results were obtained. It follows then that inhibition of the NLRP3/caspase-1/IL-1*β* pathway attenuates AHR, airway inflammatory infiltration, and mucus hypersecretion in neutrophilic asthma.

### 3.2. NLRP3/Caspase-1 Inhibitors Downregulated Inflammasome Activation and Downstream Factors

**Figure 3:**
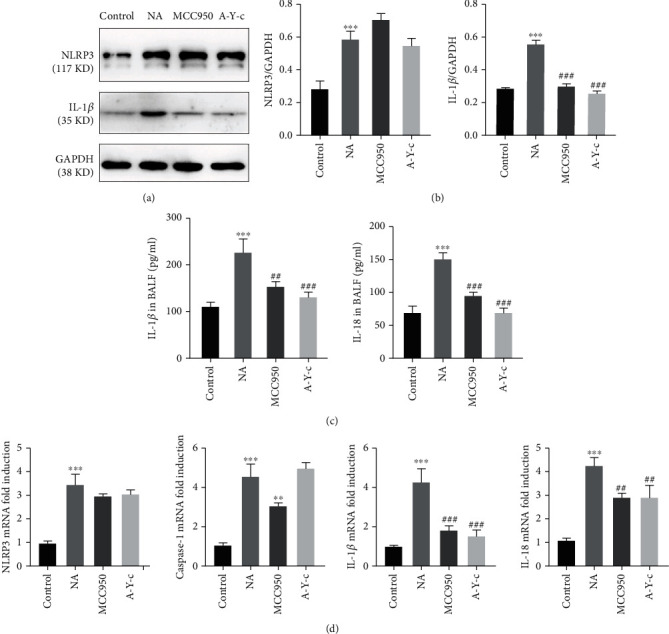
NLRP3/caspase-1 inhibitor downregulated inflammasome activation and downstream factors. (a, b) Protein expression of NLRP3 (117 KD) and IL-1*β* (35 KD) in lung homogenates was detected by western blot, and densitometric analysis was performed. (c) The concentration of IL-1*β* and IL-18 in BALF was quantified by ELISA. (d) mRNA expression of Nlrp3, caspase-1, IL-1*β*, and IL-18 in lung homogenates was detected by real-time PCR. Data are expressed as mean ± SEM. *n* = 6. Compared to the control group, ^∗^*p* < 0.05, ^∗∗^*p* < 0.01, and ^∗∗∗^*p* < 0.001; compared to the neutrophilic asthma group, ^#^*p* < 0.05, ^##^*p* < 0.01, and ^###^*p* < 0.001. NLRP3: nucleotide-binding oligomerization domain-like receptor family pyrin domain containing 3; BALF: bronchoalveolar lavage fluid; ELISA: enzyme-linked immunosorbent assay; PCR: polymerase chain reaction.

To elucidate the effect of NLRP3 inhibitor MCC950 on the activation of inflammasome, NLRP3 and IL-1*β* protein expression was measured by western blot analysis, the latter being a key downstream effector of inflammasome. As demonstrated in Figures 3(a) and 3(b), asthmatic mice's lung tissue protein levels of NLRP3 and IL-1 were much greater than controls, whereas MCC950 and the caspase-1 inhibitor Ac-YVAD-cmk almost eradicated IL-1 protein expression. The asthmatic group and the two intervention groups had similar NLRP3 expression. ELISA was used to measure IL-1 and IL-18 levels in BALF. As indicated in Figure 3(c), OVA-challenged animals showed greater levels of IL-1 and IL-18 in BALF than control mice (both *p* < 0.05), which were lowered by MCC950 and caspase-1 inhibitor.

Then, we used real-time PCR to examine inflammasome-related factor mRNA expression. The mRNA levels of NLRP3, caspase-1, and the normal downstream effectors IL-1 and IL-18 were all higher in NA lung tissue. Both NLRP3 and caspase-1 inhibitors significantly reduced IL-1 and IL-18 expression (Figure 3(d)). Moreover, MCC950 had no effect on NLRP3 transcription.

### 3.3. Blockade of the NLRP3/Caspase-1/IL-1*β* Pathway Suppressed Th17-Mediated Immune Responses in OVA-Induced Neutrophilic Asthma

**Figure 4:**
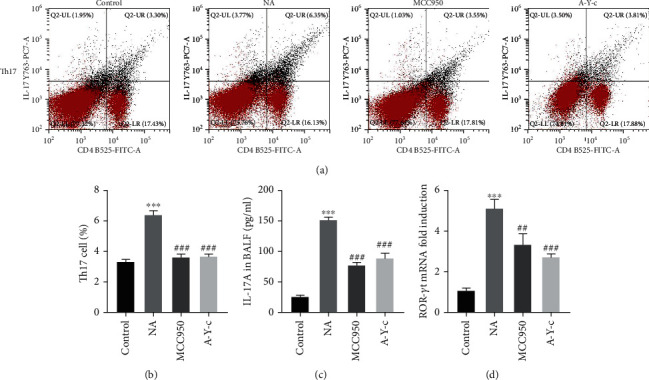
Inhibition of the NLRP3/caspase-1/IL-1*β* pathway suppressed ovalbumin-induced Th17 cell response. (a, b) Splenocytes were derived from mice and stimulated with PMA and ionomycin, and after fixed and permeabilized, cells were incubated with extracellular and intracellular antibodies. Stained cells were run on the CytoFLEX Flow Analyzer and analyzed with the CytExpert software. (c) The concentration of IL-17A in BALF was quantified by ELISA. (d) mRNA expression of ROR*γ*t in lung homogenates was detected by real-time PCR. Data are expressed as mean ± SEM. *n* = 6. Compared to the control group, ^∗^*p* < 0.05, ^∗∗^*p* < 0.01, and ^∗∗∗^*p* < 0.001; compared to the neutrophilic asthma group, ^#^*p* < 0.05, ^##^*p* < 0.01, and ^###^*p* < 0.001. NLRP3: nucleotide-binding oligomerization domain-like receptor family pyrin domain containing 3; PMA: phorbol 12-myristate 13-acetate; BALF: bronchoalveolar lavage fluid; ELISA: enzyme-linked immunosorbent assay; ROR*γ*t: retinoid-related orphan receptor-*γ*t; PCR: polymerase chain reaction.

Since the imbalance of Th17, Treg cells, and their related cytokines play a vital role in the progression of asthma [24], we investigated the effects of NLRP3 and caspase-1 inhibitors on Th17-mediated immune responses in this murine model. The fraction of IL-17A-producing CD4^+^ T cells (Th17 cells) in spleen was measured using FCM. The NLRP3 inhibitor MCC950 decreased the fraction of Th17 cells induced by OVA (Figures 4(a) and 4(b), both *p* < 0.05). We performed an ELISA to assess whether MCC950 inhibited Th17-related cytokine production in BALF. As demonstrated in Figure 4(c), the NA group had higher levels of IL-17A in BALF than the control group, although this was decreased by MCC950 therapy. The expression of the Th17 cell-related transcription factor RORt in lung tissue was also increased by OVA (*p* < 0.05) but decreased by MCC950 (*p* < 0.05) (Figure 4(d)). The caspase-1 inhibitor Ac-YVAD-cmk demonstrated comparable effects to an NLRP3 inhibitor.

### 3.4. Blockade of the NLRP3/Caspase-1/IL-1*β* Pathway Upregulated Treg-Mediated Immune Responses in OVA-Induced Neutrophilic Asthma

**Figure 5:**
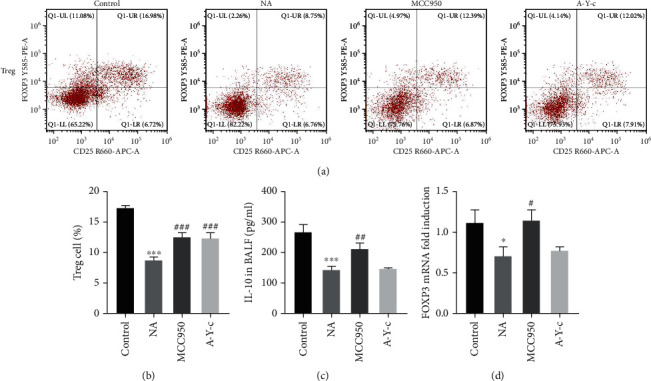
Inhibition of the NLRP3/caspase-1/IL-1*β* pathway upregulated ovalbumin-induced Treg cell response. (a, b) Splenocytes were derived from mice and fixed and permeabilized, and cells were incubated with extracellular and intracellular antibodies. Stained cells were run on the CytoFLEX Flow Analyzer and analyzed with CytExpert software. (c) The concentration of IL-10 in BALF was quantified by ELISA. (d) mRNA expression of Foxp3 in lung homogenates was detected by real-time PCR. Data are expressed as mean ± SEM. *n* = 6. Compared to the control group, ^∗^*p* < 0.05, ^∗∗^*p* < 0.01, and ^∗∗∗^*p* < 0.001; compared to the neutrophilic asthma group, ^#^*p* < 0.05, ^##^*p* < 0.01, and ^###^*p* < 0.001. NLRP3: nucleotide-binding oligomerization domain-like receptor family pyrin domain containing 3; BALF; bronchoalveolar lavage fluid; ELISA: enzyme-linked immunosorbent assay; Foxp3: fork head box p3; PCR: polymerase chain reaction.

Finally, we determined whether these inhibitors also have effects on Treg cells in this model. Figures 5(a) and 5(b) reveal that NLRP3 and caspase-1 inhibitors restored the loss of Treg cells in CD4^+^ T cells isolated from spleen. Using ELISA, we found IL-10, a Th17-related cytokine, in BALF. The content of IL-10 in the BALF of OVA-challenged mice was substantially lower than in the control group (*p* < 0.05). The decrease was due to NLRP3 and caspase-1 inhibitors (Figure 5(c)). Last, we analyzed Treg cell-related transcription factor Foxp3 mRNA expression in lung tissue. As shown in Figure 5(d), the expression of Foxp3 mRNA was also downregulated in the NA group but elevated in the MCC950 and A-Y-c groups.

## 4. Discussion

This study demonstrated that blocking the NLRP3/caspase-1/IL-1 pathway significantly reduced OVA-induced airway hyperresponsiveness, inhibited inflammatory cell infiltration into per bronchial regions, and reduced the number of inflammatory cells in BALF, while also reversing the Th17/Treg cell balance in asthmatic patients with neutrophilic airway inflammation (NAIR).

Asthma is a heterogeneous airway inflammatory disease with several clinical phenotypes, of which severe forms characterized by Th17-mediated immune responses and neutrophil-dominant airway inflammation respond poorly to corticosteroid [25, 26]. Increasing evidence links increased airway neutrophil infiltration to glucocorticoid insensitivity and decreased lung function, possibly due to lack of glucocorticoid receptor (GR) expression in neutrophils in the airway tissues. In severe asthma, a direct link between Th17 cells and airway inflammation and remodeling processes has been established [27, 28]. As previously published [29], we used an OVA-induced neutrophilic asthma mouse model with airway AHR, neutrophil infiltration, mucus hyperproduction, and increased Th17-mediated immune responses. Activation of the NLRP3 inflammasome was associated with increased expression of NLRP3, caspase-1, IL-1, and IL-18 in mice with neutrophilic asthma. As an inflammasome, NLRP3 is a multiprotein complex that has been linked to allergen-induced inflammation in the airways [30, 31]. As reported previously, NLRP3 inflammasome is mainly expressed in airway epithelial cells, T cells, and neutrophils [32]. As the first defense barrier to respond to injuries and pathogens, lung epithelial cells exhibit important protective roles in airway inflammation, while neutrophils and T cells play crucial roles in amplification of pulmonary inflammation through release of multiple inflammatory mediators [33]. Previous studies have highlighted the role of NLRP3 inflammasome in the pathogenesis of asthma. Concomitantly, a multitude of clinical and experimental studies have demonstrated that the expression of NLRP3 inflammasome is significantly increased in patients with asthma and asthmatic murine models [34, 35]. A recent research found that inhibiting the NLRP3 inflammasome reduced AHR and airway inflammation in a steroid-resistant asthma mouse model [36]. NLRP3 expression and MCC950 effects on airway inflammation in neutrophilic asthmatic mice have not been described. We investigated NLRP3 protein expression in the lungs of OVA-sensitized and challenged mice to fill this gap. The findings showed that in asthmatic mice, NLRP3 protein and mRNA expression was increased relative to the control group. In this paradigm, inhibiting NLRP3 with MCC950, a selective small-molecule inhibitor of NLRP3 inflammasome, significantly reduced OVA-induced AHR, airway neutrophilic inflammation, and mucus overproduction.

While it has been confirmed that a blockade of NLRP3 inflammasome activation alleviates neutrophilic airway inflammation, the precise mechanism involved remains poorly understood. Activation of the NLRP3 inflammasome may activate caspase-1, increasing the release of mature IL-1 and IL-18 [37, 38]. Previous research has linked NLRP3-dependent IL-1 responses to neutrophilic asthma etiology [36, 39]. Other investigations have indicated that blocking IL-1 activity with neutralizing antibodies or deleting the IL-1 receptor prevents asthma development [40]. In the OVA-induced asthma mouse model, IL-18-deficient animals had reduced neutrophilic airway inflammation and remodeling [41]. Our research found higher IL-1 and IL-18 expression in the lungs, as well as increased airway neutrophils. Inflammation caused by OVA involves IL-1 and IL-18. The NLRP3 inflammasome inhibitor MCC950 and the caspase-1 inhibitor Ac-YVAD-cmk both lowered IL-1 and IL-18 production in the lungs of OVA-exposed mice, reducing hyperresponsiveness, inflammation, and airway remodeling. The findings showed that MCC950 reduced inflammation by reducing caspase-1, IL-1, and IL-18 expression.

Neutrophil-mediated Th17 cell response has been associated to asthmatic neutrophilic inflammatory airway inflammation. Recent research shows that NLRP3 inflammasome hyperactivation increases IL-1 production, which enhances the expression of IRF4 and RORt during Th17 differentiation [42, 43]. Other research has shown that IL-1 or IL-18 in combination with IL-23 may stimulate Th17 cell IL-17 production [44]. By increasing glucocorticoid insensitivity, smooth muscle hypercontractility, and neutrophil migration to the airways, IL-17A has been shown to contribute to the pathophysiology of asthma. Decreased IL-17A production may potentially contribute to severe asthma [45–47]. Increasing the number of Th17 cells and IL-17A protein in neutrophilic asthmatic mice matched these findings. This asthmatic model's Th17 immune response was greatly reduced by inhibiting the NLRP3/caspase-1 signaling pathway with MCC950 or Ac-YVAD-cmk. Paradoxically, Foxp3^+^ Treg cells are implicated in immunological homeostasis, suppressing allergic responses, and limiting inflammation in asthma [48]. It has demonstrated that many Treg-based treatments successfully reduce allergic airway illness in several animals [49]. As revealed in our work, inhibiting NLRP3 inflammasome activation increased Foxp3 mRNA expression and the Treg immunological response. Our findings showed that inhibiting the NLRP3/caspase-1/IL-1 pathway helped rebalance Th17/Treg cells in asthma patients.

## 5. Conclusion

In a mouse model of OVA-induced neutrophilic inflammation in asthma, the current research found enhanced NLRP3 inflammasome activation. Furthermore, inhibiting the NLRP3/caspase-1/IL-1 pathway reduced AHR, inflammation, and mucus hypersecretion, while increasing Treg-mediated immune responses. While further research is needed to pinpoint the exact mechanism, these results provide light on the etiology of neutrophilic asthma and indicate that blocking the NLRP3/caspase-1/IL-1 pathway might be a viable asthma treatment target.

## Data Availability

The data used to support the findings of this study are included within the article.
